# A Matrilineal Study on the Origin and Genetic Relations of the Ecuadorian Pillareño Creole Pig Population through D-Loop Mitochondrial DNA Analysis

**DOI:** 10.3390/ani11113322

**Published:** 2021-11-20

**Authors:** Amado Manuel Canales Vergara, Amparo Martínez Martínez, Juan Vicente Delgado Bermejo, Martina Macri, Pablo Rigoberto Andino Nájera, Nelson Antonio Duchi Duchi, Paula Alexandra Toalombo Vargas

**Affiliations:** 1 Department of Genetics, Faculty of Veterinary Sciences, University of Córdoba, ceiA3, 14071 Cordoba, Spain; amparomartinezuco@gmail.com (A.M.M.); juanviagr218@gmail.com (J.V.D.B.); martinamacri@hotmail.it (M.M.); 2 Group of Research, Conservation and Management of Natural Resources of Ecuador, Escuela Superior Politécnica de Chimborazo, Panamericana Sur Km 1 1 2, Riobamba EC-060155, Ecuador; pablor.andino@espoch.edu.ec (P.R.A.N.); nelson.duchi@espoch.edu.ec (N.A.D.D.); ptoalombo@espoch.edu.ec (P.A.T.V.)

**Keywords:** DNA D-loop, creole pigs, Tajima’s D, separated

## Abstract

**Simple Summary:**

Mitochondrial DNA (mtDNA) analysis is a tool in assessing the maternal origin, phylogeny, and population structure of domestic animals. The Ecuadorian Pillareño Creole pig is a creole breed that comes from the descendants of Iberian pig introduced to Ecuador by the Spanish conquerors. This creole population represents important reservoirs of genetic diversity that are very essential to preserve; however, the introduction of new breeds of pigs has displaced this creole pig from Ecuador to the background. The objective of the mitochondrial DNA analysis was to identify the Ecuadorian Pillareño Creole pig populations, their origins, and their maternal lines. For this study, DNA samples from 34 Ecuadorian Pillareño Creole pigs were used, with the animals belonging to seven rural regions of Ecuador. The haplogroup network suggested that the Pillareño creole pig population can be grouped into a single haplotype and that it belongs to the European pig clades. The genetic relationship between the Ecuadorian Pillareño Creole and the European pigs, particularly the Iberian pigs from Spain, can be used to establish of an official breeding program for the conservation and valuation of these creole populations, with this genetic mitochondrial analysis potentially providing a better approach for the rescue of the Ecuadorian Pillareño Creole pig populations.

**Abstract:**

Domestic pig breeds reached America on the second Columbus trip; from this date, Iberian pig genetic resources were disseminated throughout the continent, forming diverse creole breeds. These Ecuadorian Creole pigs are important for food production but have been genetically eroded since the introduction of transboundary breeds. In this study, we sought to characterize this erosion more thoroughly through mitochondrial DNA D-Loop analysis of Ecuadorian Pillareño Creole pigs from seven regions of Ecuador. To allow comparison, we also included in our analysis sequences from wild species, commercial lines, and domestic pigs, which were obtained from the NCBI GenBank database. Creole pigs’ population showed overall moderate Hd values and low π values, and a negative value of Tajima’s D was observed. The greatest differentiation from the Ecuadorian Pillareño Creole pigs was observed between Asian wild and Asian domestic pigs. The haplotype analysis revealed three different phylogenetic clades (A, E I, and E II) and 65 haplotypes. Ecuadorian Creole populations were grouped into nine haplotypes for Clade E I and E II, which have not previously been reported for Creole Pillareño populations. Our analysis indicates that in the establishment of Creole Pillareño pigs, individuals most likely separated from the Asian pig population and appear to be genetically influenced by European and Iberian populations raised in Spain.

## 1. Introduction

Domestic pig colonization of the American continent started with the second trip of Columbus; in this expedition, several domestic species were carried to the Caribbean islands. Among them, pigs had a special role because the meat was used to feed the population and to prevent vitamin C deficiency [1]. From the Caribbean archipelago, the pig resources multiplied and were distributed in three main directions. The first was to North America, reaching the north of Mexico and the present southwestern United States. The second was to the south of Peru, reaching the Patagonia region. The third was to Central America through the Atlantic ports of Panama and Cartagena de Indias, Colombia, reaching the south of Mexico and the northern countries of South America, including Ecuador. This migration resulted in the local domestic breeds present in the different territories; these pig breeds are currently known as “Creole pigs”. Some feral populations also developed, which remain in several countries such as Argentina, Brazil, and Uruguay, among others.

Today, different populations of these Creole pigs exist in America, for instance, in Colombia (San Pedreño and Zungo pig), Mexico (Pelon mexicano pig), Uruguay (Pampa Rocha pig), Argentina (Chancho Cimarrón pig), Cuba (Pinareño Creole pig, Cuban Creole pig), Venezuela (Apure Creole pig), Peru (Tumbes Creole pig), Guatemala (Chorti Creole pig), Brazil (Piau pig, Porco monteiro), and the United States (Ossabaw, Choctaw, Mulefoot, and Red Waddler pig), among others [2,3,4,5], but many more remain unknown, and studies characterizing these populations are needed. 

In Ecuador, the Pillareño Creole pig populations persist; these animals are normally under poor management conditions in marginal areas, generally involving family backyard production [6]. For more than 500 years, the Ecuadorian Pillareño Creole pigs have adapted to very different conditions in Ecuador and demonstrate climatic resistance [7], with a great adaptability to different ecosystems (Andinian, Coastal, and Amazonian), especially to extremely adverse conditions and to a diet with a low nutritional level [8]. Alongside the important social role of the Ecuadorian Pillareño Creole pigs, these animals have great potentiality for profitable and sustainable production due to their putative descendance from the Iberian pig, and probably carry its exceptional meat quality characteristics. 

Unfortunately, it was verified that in Ecuador, breeds of Iberian origin have tended to disappear due to the aggressive introduction of commercial breeds from northern European countries, which puts at risk a genetic heritage worthy of being conserved in order to take advantage of these capabilities, such as resistance to diseases, rusticity, and ability adaptive capacity to different environments [9].

Historical documents illustrate that the Spanish conquerors released different lineages of the Iberian pig (Smooth Black, Hairy Black, Red Dish, Dark Brown, and Andalusian Blonde) [10] in America. Phylogeographical studies of domestic animals are based on finding genetic variations in mitochondrial DNA (mtDNA) because its variability is five times higher than for other types of markers [11,12]. The mtDNA structure shows nonrecombining patterns in pigs, forming a closed circular double helix DNA sizing around 16,500 bp encoding 13 hydrophobic polypeptides, 22 tRNAs, and 2 rRNAs [13,14].

Generally, the research of genetic distances between breeds due to huge mutations [15] based on genetic variants in mtDNA has been centered on the D-loop region. This information is also available to characterize breeds and individuals in phylogenetic studies [16]. However, whole mtDNA sequences are needed to estimate the genetic relationships among breeds, characterize breed specificity, and identify individuals.

Different American, European, and Asian pig breeds have been studied at the molecular level using diverse nuclear DNA marker systems [17,18], including microsatellites and single nucleotide polymorphisms (SNPs), but the relationships among Creole pigs have not been extensively evaluated using the mitochondrial DNA (mtDNA) D-loop control region. In terms of the possibilities of this molecular tool [13], mtDNA assessment can explain and provide additional support for the evaluation of distinctions between the Ecuadorian Pillareño Creole, European, Iberian, Asian, and commercial pig breeds on the basis of their relatedness [19,20].

In the present paper, we investigated the Ecuadorian Pillareño local pig in terms of its possible Iberian origin and recent genetic erosion, understanding it as a loss of biodiversity due to crossbreeding with transboundary breeds. This was achieved by analyzing the mitochondrial diversity in Ecuadorian Pillareño Creole pig and testing its matrilineal relations with European domestic lines, Iberian pigs raised in Spain, Asian pig lines, commercial pigs, and wild pig breeds to evaluate its origin and inter- and intraspecific global connections with these other pig populations.

## 2. Materials and Methods

### 2.1. Sample Collection

According to the livestock census, as well as the creole pig Pillareño morphological and phenotypic measures, we selected backyard pigs raised by families in several locations throughout Chimborazo. A total of 34 blood samples of Creole Pillareño pigs were collected and preserved in 5 mL tubes containing ethylenediaminetetraacetic acid (EDTA). In order to avoid a high relationship between the animals, we collected the samples in seven different regions in Ecuador. For each region (Pungalá, Tunshi, Molobog Licto, Licto Pungala, Guamote, Pulinguí, and Penipe), five animals were selected, apart from Penipe, where there were only four animals. After the samples were obtained, they were preserved and stored at −18 °C for DNA extraction in the Animal Breeding Consulting Laboratory located at University of Córdoba, Spain, until subsequent use.

### 2.2. Ethics Statement

This type of project does not fall under the legislation for the protection of animals used for scientific purposes, Organic Law for the Defense of Animal Rights. Data were collected during the application of regular zootechnical procedures without injuring the animals.

### 2.3. DNA Extraction and Amplification

DNA was extracted from blood samples by using a Chelex-100 resin^®^ analytical grade, 50–100 mesh, sodium form (BioRad, Madrid, Spain) under the protocol used by [1]. The mtDNA D-loop sequence was obtained from GenBank accession number AJ002189 [2] and primers (F: 5′-CGCCATCAGCACCCAAAGCT-3′ and R: 5′-TGGGCGATTTTAGGTGAGATGGT-3′) [21,22,23] to amplify a 637 bp product from [3,19,20,22,23] the mtDNA region. The reactions were performed following the steps by Canales [4]. The amplicon quality of the product of PCR were assessed in a 1.5% agarose gel stained with ethidium bromide (low EEO/multifunctional/molecular biology grade) using size 100 bp DNA ladder^®^ (Invitrogen, Waltham, MA, USA) as a banding marker. After verifying the PCR product, the reactions were incubated using 10 µL with one unit of FastaP Thermosensitive Alkaline Phosphatase (Thermo Fisher Scientific, Waltham, MA, USA) and 10 units of Exonuclease enzyme (Thermo Fisher Scientific, Waltham, MA, USA) incubated by one cycle at 37 °C for 15 min and 80 °C for 15 min. The sequencing reactions were performed in both directions using PCR oligos by the dideoxy technique, using a commercial service at Macrogen Inc. services (Madrid, Spain). In addition, we included sequences from wild species (*Sus celebensis* indonesia*, papuensis vanuatu, barbatus,* and *wild Spanish*), many commercial lines, and domestic pigs (China, Indonesia, Papua New Guinea, Germany, Italy, Malaysia, France, Iberian, Black Jabugo, Large White, Duroc, and Pietrain), obtained from the NCBI GenBank database, resulting in a total of 134 sequences. More details about the country of origin and the number of samples for each breed are given in the electronic supplementary material (Table S1).

### 2.4. Molecular D-Loop Analysis

#### 2.4.1. Mitochondrial Genetic Diversity and Differentiation

The sequence editing, alignment, and construction of data matrices were performed using Mega v5 [5] and Gblocks 0.91b [6,7]. All new sequences were deposited in GenBank. The number of haplotypes (H) and polymorphic sites (S), amount of nucleotide diversity (π) and haplotype (Hd) diversity estimates, and the calculation of Tajima’s D-values Fu’s and Fs statistics for the Ecuadorian Creole pig populations were calculated using DnaSP v5 [7]. In addition, F_ST_ and coancestry coefficient (permuting haplotypes among population among groups) values from pairwise comparisons were computed with 5000 permutations using ARLEQUIN v3.1 [8]. A neighbor-joining (NJ) tree was constructed on the basis of the genetic distance matrix using Splitstree v4.14.6 software [9]. Analysis of molecular variance (AMOVA) [10] was used to calculate genetic variation and differentiation between populations by performing 10,000 permutations. We included the following external population sequences from the NCBI GenBank database: the Iberian-like population (Torbiscal line, Black Hairless, Red, Black Hairy, and Blond); wild species from Asia (Sus celebensis indonesia, papuensis vanuatu, and barbatus); wild Spanish pig, domestic pigs from Asia (China, Indonesia, Papua New Guinea, and Malaysia); European pigs from Germany, Italy, and France; and different commercial lines from Europe (Black jabugo, Large White, Duroc, and Pietrain).

#### 2.4.2. Genealogical Relationships between Haplotypes

With the software NETWORK v4.6.0.0 [9], we constructed a haplotype network to establish genealogical relationships between haplotypes and their frequencies using the median-joining method under the default parameters. We analyzed the relationship between haplotypes and sequence variation using phylogenetic inference. The matrices included haplotypes that were identified in this study and haplotypes for each population that were available from the NCBI GenBank database. The network consisted of 134 frequencies and included wild species (*Sus celebensis* indonesia*, papuensis vanuatu, barbatus,* and *wild Spanish*), many widely distributed commercial lines, local domestic pig breeds (China, Indonesia, Papua New Guinea, Germany, Italy, Malaysia, France, Iberian, Black Jabugo, Duroc, and Pietrain), and the Ecuadorian Creole pig (Table S1).

## 3. Results

### 3.1. Sequence Analysis, Genetic Diversity, and Differentiation

After we amplified the 637 bp product from [3] the mtDNA region, 34 sequences were edited and aligned, and 550 bp of the mtDNA D-loop was obtained from DNA samples of Pillareño pigs from Ecuador collected for this study. These sequences were registered in GenBank (accession numbers: MT317953–MT317986). D-loop sequences were aligned to a reference sequence from GenBank (accession number AJ002189); nine haplotypes with 25 polymorphic sites were identified in the population of Pillareño. The dominant haplotype was H_3 with *n* = 21 pigs (Table 1). All the populations showed overall moderate Hd values and low π values, with a negative value of Tajima’s D [11], which indicates an excess number of alleles from a recent population or genetic hitchhiking, with the Fu’s Fs tests showing positive values. All the results are shown in Table 2.

**Figure 1:**
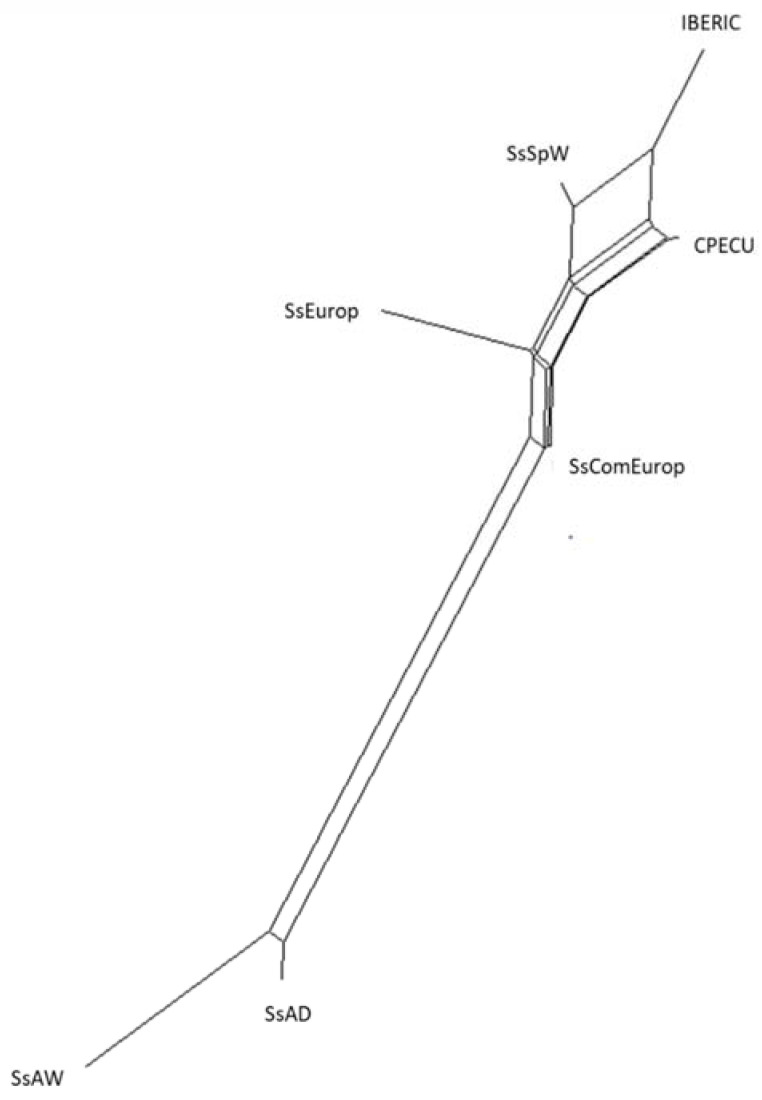
Neighbor-net graph drawn by different populations and 134 pig mitochondrial sequences studied by using the splits tree 4.0 program. CPECU: Pillareño; SsEurop: European domestic pigs; IBERIC: Spanish Iberic pigs; SsComEurop: Commercial European pigs; SsAD: Asian domestic pig; SsSpW: Spanish wild pig; SsAW: Asian wild pig.

In addition, we analyzed and constructed one genetic differentiation table, Table 3, which shows that the main divergence of Pillareño was observed between Asia domestic and Asia wild, and the lowest rates of genetic divergence were found between Pillareño and Iberic, Spanish wild, and commercial European. To confirm our results, using the neighbor-joining method (Figure 1), we estimated the genetic distances between populations from mitochondrial sequences.

The coancestry coefficients [12] were calculated, and the greatest coefficients were observed with Pillareño–Asian domestic pigs; by contrast, the lowest genetic coancestry coefficients differentiation values were found between Pillareño and Iberic pigs (Table 4)

The total amount of genetic variation detected was partitioned by AMOVA (*p* < 0.01), according to the following groups: Pillareño vs. European, Iberic, and commercial. According to the previous components divided by region and populations, the analysis results in Table 5 show that the genetic variability within population components was 69.42% of the total variance. A significant amount of within-group variation was also observed (28.36%), and a smaller but still significant result of difference among groups (2.22%) was found, with the high fixation index (F_SC,_ F_ST_ and F_CT_) indicating that populations were well differentiated.

### 3.2. Haplotype Network

**Figure 2:**
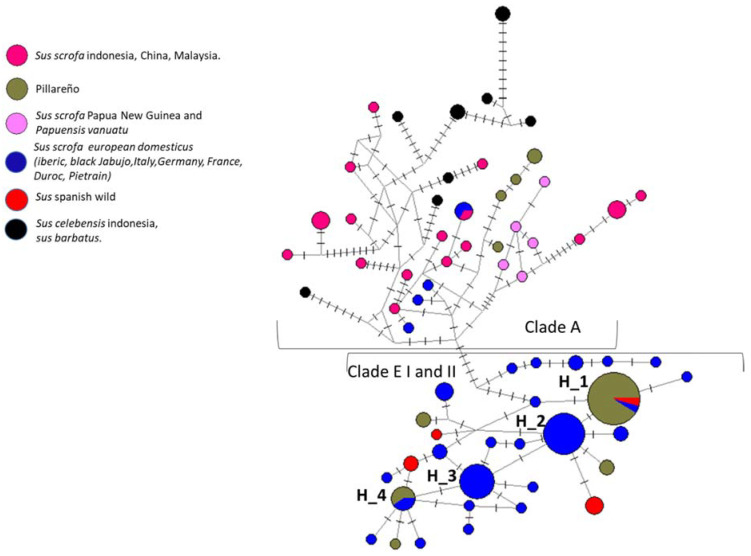
Median-joining haplotype network. Circles represent haplotypes identified in different clades; areas of the circles are proportional to frequency, and colors represent the populations, including Asia, Pillareño, European, and wild individuals.

We constructed a haplotype network to visualize the relationships between haplotypes and their frequencies, and the study showed that they are distinguished from each other by a moderate number of mutations. The network (Figure 2) showed and separated very clearly three different phylogenetic clades (A, E I, and E II) and 65 haplotypes. In the clade A, all the Asian domestic and wild pig haplotypes were grouped; in this clade, it was possible to observe four unique haplotypes for the Ecuadorian Creole pig.

The other haplotypes conformed to the E I phylogenetic clades that corresponded to the European and E II that conformed to wild pig from Europe [13,14]. In these two clades, it was possible to observe that the majority of the sequences were of Pillareño; the network (Figure 2) displayed four principal haplotypes (H_1, H_2, H_3, and H_4). H_1 was the main haplotype and was composed of 23 individuals and three haplogroups. The dominant haplogroup corresponded to the Pillareño Creole pig samples, and the other two haplogroups corresponded to Iberian pigs and Spanish wild pigs. In the periphery of these haplotypes, we observed that they were surrounded by unique sequences of European domestic pigs and the haplotype H_2. H_2 is related to H_1 and was formed by the Iberian domestic pig haplogroup. The H_3 haplotypes contained European domestic pig samples. Haplotype H_4 comprised two haplogroups, which consisted of three individuals belonging to Pillareño Creole pig and two European individuals (Iberian and Duroc). H_2, H_3, and H_4 presented a star-like profile, consistent with the pattern of population expansion in the past.

## 4. Discussion

There is a new perspective regarding the use of adapted local breeds in programs of rural development that involves important concepts of sustainability, food security, and food sovereignty; even so, the Creole pigs are in extreme danger of extinction, owing to a severely reduced population. Ecuador is a good example in that this country has developed important policies for the economic growth of the poorest regions with Afro-American and indigenous populations. These recent programs have involved chickens [15], goats [16], and pigs [17].

In this study, we present the first results to understand the evolution of the population of Pillareño Creole pig; the analysis suggests a moderate level of haplotype diversity (0.615) that represents the probability that two randomly sampled alleles are different, while the low nucleotide diversity (0.00968) represents the average number of nucleotide differences per site according to the analysis of generated sequences [18,19]. These two results are Hd and π, indicators that the Pillareño Creole pig population originated from a small number of founders [20], showing that the populations had in their evolution the effect of a bottleneck, producing genetic drift or changes in genetic sequences from the ancestors in Europe. These results differed from those reported for Alves [21], wherein pig populations of Iberian and maternal lineages preserved in the Torbiscal line were characterized and showed six mtDNA and 12 mtDNA haplotypes, respectively, but similar results reported in Mexican Pelon Creole pig [22] showed nine haplotypes. The negative value of Tajima’s D indicated an excess of rare haplotypes over what would be expected under neutrality and a signature indicating recent population expansion. Hence, the positive Fu’s Fs test results signified low levels and high frequency polymorphisms, indicating a recent decrease in population size and balancing selection [23]. These findings support the origin of the Pillareño Creole pig in those animals brought by the Spaniards in the conquest of Ecuador, but the sequential bottleneck produced a differentiated mitochondrial DNA profile.

In the colonization of America by domestic animals, pigs were incorporated early because these animals were carried on the boats to prevent scurvy due to vitamin C deficiency through use of pig meat as a protein supply of animal origin [24].

In the Table 3, is possible to observe that the Pillareño Creole and all the Asian populations showed genetic differences and variation with the European population, and this was possible because the Iberian populations contributed to the development of the Pillareño Creole [25]. It is also possible to observe the low mitochondrial genetic differences, assuming the complete influence of these populations in the Pillareño Creole pigs [26,27]; at the same time, the Spanish wild population showed a low-to-moderate distance from the Pillareño Creole population, likely because they mated with the European wild boar. Phoenician pigs are mentioned in historical reports as having been brought to southern Europe and the Iberian Peninsula by the Romans, and continued attempts to improve the husbandry and breeding of the native pigs contributed to the rise of the modern Iberian pig [28]. The coancestry coefficients (Table 4) are useful because they show how much the genomes of two individuals are expected to resemble each other [12], and the F_ST_ results for Pillareño Creole pig showed this population was preserved and was not influenced by commercial populations. This information was inferred from the neighbor-net results obtained (Figure 1). This is evidence for the purity of this breed in spite of the pressure from commercial lines of foreign breeds, and therefore this pressure has produced, at least at the maternal level, a reduction in the Pillareño population but not an indiscriminate crossbreeding with exotic breeds.

In the genetic differentiation and the distribution of genetic variation by the AMOVA analysis, genetic differences were observed within populations, indicating that the populations of European, Pillareño Creole, and Iberian pigs shared common haplotypes and a maternal lineage, and it was also observed that the variation of mtDNA between populations and the proportion of unshared haplotypes in each group was significant and variable [29,30]. This supports the historical theories about the primary origin of the Latin American Creole pig populations.

Very few studies of mtDNA have been performed in Creole pig populations; it is worth mentioning that we were able to find a distinctive Iberian genetic signature in all the Ecuadorian Creole pig haplotypes. This finding might be explained by the fact that the Iberian populations had a strong phylogeographic structure at the time of American colonization [31]. The population of this study was detected in the clade E I and E II, corresponding to the Iberian clades [21], and with clear evidence of separation from the Asian populations, with the exception of five individuals that presented a haplotype in Clade A.

Accordingly, the presence of this individual in the Asian clade could be a possible explanation for the introgression of common Asian commercial lineages associated with Clade A or the historical influence of Philippine pigs [27] introduced more recently from their colonies in Asia by the Spaniards, shipped in the so-called “Navío de Manila”, which navigated the Pacific Ocean from the Philippines to the Pacific coast of America. The majority of Ecuadorian Creole individuals were of the principal haplotype. They shared haplogroups with wild Spanish individual and Iberian pigs, having a matrilineal relationship, because the Iberian pigs are currently genetically interacting with the Spanish wild boar, and for this reason the wild Spanish pig shares this haplotype [28].

## 5. Conclusions

All analyses and the expected level of genetic similarity of mtDNA showed moderate haplotype diversity and low nucleotide diversity among the Pillareño Creole pig population and indicate that the populations of Creole pigs from Ecuador have directly descended from an Iberian ancestral population from Spain. Over the time, these populations then thrived and became stable with the correct animal breeding, and this genetic inheritance has been preserved throughout the years.

The evolutionary linkage of the Pillareño Creole pig with the Iberian pig suggests the population is an important source of sustainable richness and has the genetic base to follow the Iberian pig model of production according to the quality of the products and with respect for the environment.

Pillareño Ecuadorian Creole populations from rural communities in Ecuador were grouped in nine haplotypes with Clade EI and EII, which have not previously been reported for Creole populations; hence, this study could be considered a model for future research into other Creole pig populations of the American continent.

## Figures and Tables

**Table 1:** Variable positions in mtDNA D-loop in Pillareño Creole pig (CPECU). Sequence identities (“.”) and deletions are indicated by dots and dashes. Nucleotide positions are numbered according to the reference sequence GenBank AJ002189 [2].

	Nucleotide Positions																									
Haplotypes							1	1	1	1	1	1	1	1	2	2	2	2	2	2	3	4	4	4	5	5
		3	6	6	8	8	0	0	1	3	3	7	7	9	3	3	5	5	6	8	4	0	0	5	1	3
		3	5	6	0	7	1	9	4	7	8	0	1	7	6	9	1	8	3	0	7	0	9	8	7	2
	AJJ002189	C	T	G	A	G	C	C	A	T	T	T	T	T	T	C	A	C	C	T	C	A	C	A	T	A
H_1	CPECU: 31	.	C	.	.	A	T	T	G	C	C	C	.	C	C	.	G	.	T	.	T	.	T	G	C	G
H_2	CPECU: 13, 21	T	.	A	.	.	.	.	.	.	.	.	.	.	.	.	.	.	.	C	.	.	.	.	.	.
H_3	CPECU: 39, 11–12, 15, 17, 19, 20, 22, 24, 27, 29, 30, 32	.	.	.	.	.	.	.	.	.	.	.	.	.	C	.	.	.	.	.	.	.	.	.	.	.
H_4	CPECU: 33	.	C	.	.	A	T	T	G	C	C	C	.	.	C	.	G	.	T	.	T	.	T	G	C	G
H_5	CPECU: 23	.	C	.	.	A	T	T	G	C	.	.	.	.	C	.	G	T	T	.	T	.	T	.	C	G
H_6	CPECU: 28	.	.	.	T	.	.	.	.	C	.	.	.	.	C	T	.	.	.	C	.	.	.	.	.	.
H_7	CPECU: 2, 14	.	C	.	.	A	T	T	G	C	C	C	A	C	C	.	G	.	T	.	T	.	T	G	C	G
H_8	CPECU:1, 16, 18	.	.	.	T	.	.	.	.	C	.	.	.	.	C	.	.	.	.	C	.	.	.	.	.	.
H_9	CPECU: 10, 24	.	.	.	.	.	.	.	.	.	.	.	.	.	C	.	.	.	.	C	.	G	.	.	.	.

**Table 2:** Genetic diversity indices for Pillareño Creole pig population.

Population	Genetic Diversity Indices						Neutrality Test	
	N	Tnm	H	Hd	π	S	Tajima’s D (p)	Fu’s FS
Pillareño Creole pig	34	25	9	0.615	0.00968	25	−0.44963 (0.454)	1.794 (0.119)

N: number of individuals; Tnm: total number of mutations; H: number of haplotypes; Hd: haplotype diversity; π: nucleotide diversity; S: number of polymorphic sites. FS: F statistical.

**Table 3:** Pairwise genetic differentiation (F_ST_—permuting haplotypes among population among groups) of populations.

Pig Population	CPECU	SSEUROP	IBERIC	SSCOMEUROP	SSAD	SSSPW	SSAW
CPECU	0.00000						
SSEUROP	0.19955	0.00000					
IBERIC	0.11760	0.25758	0.00000				
SSCOMEUROP	0.12242	0.14175	0.27297	0.00000			
SSAD	0.47947	0.45415	0.56992	0.31056	0.00000		
SSSPW	0.12439	0.19741	0.12655	0.13017	0.47835	0.00000	
SSAW	0.58502	0.55411	0.66240	0.42897	0.15217	0.56351	0.00000

CPECU: Pillareño; SSEUROP: European domestic pigs; IBERIC: Spanish Iberic pigs; SSCOMEUROP: Commercial European pigs; SSAD: Asian domestic pig; SSSPW: Spanish wild pig; SSAW: Asian wild pig.

**Table 4:** Coancestry coefficient indices for each population studied.

Pig Population	CPECU	SSEUROP	IBERIC	SSCOMEUROP	SSAD	SSSPW	SSAW
CPECU	0						
SSEUROP	0.22258	0					
IBERIC	0.12511	0.29784	0				
SSCOMEUROP	0.13059	0.15286	0.31879	0			
SSAD	0.65291	0.60542	0.84378	0.37188	0		
SSSPW	0.13284	0.21991	0.13531	0.13945	0.65075	0	
SSAW	0.87952	0.80769	1.08588	0.56031	0.16508	0.82900	0

CPECU: Pillareño; SSEUROP: European domestic pigs; IBERIC: Spanish Iberic pigs; SSCOMEUROP: Commercial European pigs; SSAD: Asian domestic pig; SSSPW: Spanish wild pig; SSAW: Asian wild pig.

**Table 5:** Summary of the results of analysis of molecular variance (AMOVA), with significant value (*p* < 0.01) without a priori assumptions defined by population.

Source of Variation	Df	Sum of Squares	Variance Components	Percentage of Variation	Fixation Indices
Among groups	3	47.108	0.06985 Va	2.22	
Among populations/within groups	9	55.027	0.89171 Vb	28.36	F_SC_: 0.29004 F_ST_: 0.30581 F_CT_: 0.02221
Within populations	84	183.350	2.18274 Vc	69.42	
Total	96	811.794	3.14430		

Group: Pillareño vs. Europeans, Iberic, and commercial. Variance for group, Va; variance for population, Vb; variance for haplotypes within a population within a group, Vc; permuting haplotypes among population among groups, F_ST_; permuting haplotypes among populations within groups, F_SC_; permuting populations among groups, F_CT_. Df; Degrees of freedom.

## Data Availability

https://www.ncbi.nlm.nih.gov/popset?DbFrom=nuccore&Cmd=Link&LinkName=nuccore_popset&IdsFromResult=2049649592, (accessed on 17 November 2021).

## Supplementary Materials

The following are available online at https://www.mdpi.com/article/10.3390/ani11113322/s1, Table S1: Access code obtained from GenBank of the sequences used in the study.
